# Development of Microsatellite Markers for the Korean Mussel, *Mytilus coruscus* (Mytilidae) Using Next-Generation Sequencing

**DOI:** 10.3390/ijms130810583

**Published:** 2012-08-22

**Authors:** Hye Suck An, Jang Wook Lee

**Affiliations:** Biotechnology Research Division, National Fisheries Research and Development Institute, Busan 619-705, Korea; E-Mail: lee9952@nfrdi.go.kr

**Keywords:** Korean mussel, *Mytilus coruscus*, microsatellite loci, next-generation sequencing, genetic variability

## Abstract

*Mytilus coruscus* (family Mytilidae) is one of the most important marine shellfish species in Korea. During the past few decades, this species has become endangered due to the loss of habitats and overfishing. Despite this species’ importance, information on its genetic background is scarce. In this study, we developed microsatellite markers for *M. coruscus* using next-generation sequencing. A total of 263,900 raw reads were obtained from a quarter-plate run on the 454 GS-FLX titanium platform, and 176,327 unique sequences were generated with an average length of 381 bp; 2569 (1.45%) sequences contained a minimum of five di- to tetra-nucleotide repeat motifs. Of the 51 loci screened, 46 were amplified successfully, and 22 were polymorphic among 30 individuals, with seven of trinucleotide repeats and three of tetranucleotide repeats. All loci exhibited high genetic variability, with an average of 17.32 alleles per locus, and the mean observed and expected heterozygosities were 0.67 and 0.90, respectively. In addition, cross-amplification was tested for all 22 loci in another congener species, *M. galloprovincialis.* None of the primer pairs resulted in effective amplification, which might be due to their high mutation rates. Our work demonstrated the utility of next-generation 454 sequencing as a method for the rapid and cost-effective identification of microsatellites. The high degree of polymorphism exhibited by the 22 newly developed microsatellites will be useful in future conservation genetic studies of this species.

## 1. Introduction

*Mytilus coruscus* is one of the most favored commercially important shellfish species among approximately 20 species of mussels in Korea, which inhabits the coastal areas of Eastern Asia, including Korea, Japan and China [1]. Recently, this species has become endangered as a consequence of overfishing and/or the loss of habitats due to competition with an invasive species, *M. galloprovincialis* [2,3]. This invasive species is believed to have been carried in the ballast tanks of ships from western European ports and is now the dominant cultivated species in Korea [2,3]. The decline in the *M. coruscus* catch has increased interest in the genetic characteristics of this mussel species with the goal of developing a sustainable fishery. Knowledge regarding the genetic variability and the patterns of the stock structure is a prerequisite for developing effective fishery conservation strategies, management and remediation efforts [4]. Despite the strong commercial interest in the mussels in Korea, to date, the lack of robust polymorphic molecular markers has limited studies on their genetic background.

Among the various currently available DNA markers, microsatellites, also known as simple sequence repeats (SSRs), are very useful molecular markers because they have a number of desirable features, such as ease of use, codominance and high mutation rates [5]. Microsatellite DNA markers have been used extensively to detect genetic diversity and to evaluate population structure in marine organisms, including shellfish species [6–8]. However, few microsatellite markers have so far been published for *M. coruscus*. Recently, 12 microsatellite markers for this species were characterized from expressed sequence tag (EST)-cDNA sequences and an enriched genomic library, but these markers were found to have relatively few alleles, with nine loci having fewer than five alleles each and most of them are dinucleotide repeats [9]. Thus, the number of microsatellites for the Korean mussel is too small to conduct further population genetic analyses, including assignment tests, pedigree analysis, and mapping studies. In addition, the tri- and tetranucleotide microsatellites are reported to be more polymorphic and stable than the dinucleotide ones [10]. Therefore, the development of powerful and efficient species-specific markers is necessary to analyze the population genetics of this endangered species.

The traditional process of developing genomic microsatellite markers is time consuming and expensive due to the preparation of genomic libraries and the subsequent sequencing of a large number of clones that potentially contain microsatellite regions [11–13]. However, recently developed next- generation sequencing platforms, such as the 454 GS-FLX platform (Roche Applied Science), facilitate high-throughput genome sequencing and provide a much more efficient and cost-effective method for the acquisition of genetic markers, including microsatellites, in those organisms for which adequate databases are not currently available [14,15].

In this study, we developed 22 novel polymorphic microsatellite primer sets using 454 GS-FLX pyrosequencing. The polymorphic microsatellite markers described here will be useful for future genetic studies to understand the genetic status and facilitate the conservation of the endangered species *M. coruscus* in Korea. Additionally, the applicability of these markers in another congener species was evaluated via cross-species amplification experiments.

## 2. Results and Discussion

### 2.1. 454 Sequencing Results

The raw sequence data from a quarter-plate run of 454 sequencing included 104 Mbp containing 263,900 reads or sequences with an average length of 392 bp (maximum: 766 bp, minimum: 40). The raw sequences could be assembled into contigs. This process eliminates the repetitive sequences and creates longer reads, which may increase the probability of detecting microsatellite repeats and suitable primers within a read [16]. A total of 68,841 reads (approximately 26.1%) were assembled into 9795 contigs with an average length of 391 bp (maximum: 11,362 bp, minimum: 100 bp), leaving 166,532 singletons. The mean length of these 176,327 sequences (9795 contigs plus 166,532 singletons) was 381 bp which was similar to that of the raw sequences. Of the 176,327 unique sequences, 2569 (1.45%) sequences contained a minimum of five di- to tetra-nucleotide repeat motifs for suitable use as polymorphic microsatellite markers. Five to six repeat motifs were the most abundant type of repeats (75.3%), followed by seven to nine repeat motifs (19%) and greater than ten repeat motifs (5.7%).

### 2.2. Microsatellite Loci Isolation

Of the 2569 sequences containing a minimum of five repeats motifs, 147 sequences with a minimum of ten di- to tetra-nucleotide repeat motifs were used to develop microsatellite primers. To design, the primers, those sequences that exhibited adequately long (more than 400 bp) and unique sequence regions flanking the microsatellite array (minimum 100 bases) were selected. Thus, 51 microsatellite loci (29 di-, 12 tri- and 10 tetra-nucleotide) were selected for subsequent polymorphism screening. Of these 51 microsatellite loci, 46 (90.2%; 25 di-, 11 tri- and 10 tetra-nucleotides) loci were amplified successfully on an agarose gel for the initial evaluation of the microsatellite primers. The remaining five primers did not generate the desired amplification products in all the eight samples despite retests under modified PCR conditions. Additionally, ten loci showed faint or inconsistent bands, which may be due to nonspecific PCR amplification. Subsequently, further screening revealed that 22 (47.8%) loci were polymorphic in the eight *M. coruscus* samples. The primer sequences, repeat motifs, annealing temperatures, fluorescent labels and GenBank accession numbers for the 22 new microsatellite loci are summarized in Table 1.

Understanding the genetic diversity of *M. coruscus* populations is vital for stock abundance recovery and the planning of sustainable fishery management. To this end, microsatellite markers have overtaken mitochondrial and other DNA markers that are currently employed. The introduction of microsatellite markers to population genetic studies has greatly advanced our ability to determine the population genetic structure, test parentage and relatedness, assess genetic diversity and study recent population history [17]. Hence, Xu *et al.* [9] developed 12 polymorphic microsatellites using an expressed sequence tag-library and two microsatellite-enriched genomic libraries for *M. coruscus*; the allelic number of alleles ranged from three to seven in the tested population. In this study, 2569 sequences containing a minimum of five repeats motifs were detected for this endangered species, and 22 polymorphic microsatellite loci were developed from 51 sequences containing at least ten repeats motifs and with a minimum length of 400 bp, including a minimum of 100 bases flanking the microsatellite array. Twelve polymorphic microsatellite loci were dinucleotide repeats, seven trinucleotide repeats and three tetranucleotide repeats. At present, for most fish species for which microsatellite markers have been developed, dinucleotide repeats such as (CA)*_n_* are still the predominant markers. Tri- and tetranucleotide microsatellites, however, have the advantage over dunucleotides of being highly polymorphic and more stable and presenting clearer bands [10,18]. The advantages of generating microsatellites by pyrosequencing were realized in this study. One advantage is that microsatellite loci can be located rapidly and with decreased costs. The better advantage is that many loci are detected without any enrichment which enable to screen all the repeat motifs present, allowing the targeted selection of the loci that are most likely to amplify and be polymorphic. Although genomes vary substantially in their frequency of microsatellites, the number of microsatellites detected is most likely inflated by the occurrence of multiple reads covering the same sequence [16].

### 2.3. Genetic Characterization

Samples of 30 natural *M. coruscus* collected from Taean, Korea, were screened for variation at the 22 new polymorphic microsatellite loci. The 22 primer sets yielded variable profiles. Reruns were conducted for 53.3% of all individuals to ensure allele scoring reproducibility. The statistical results for the 22 new microsatellite loci are summarized in Table 1. A homology search using the program BLAST showed that none of the 20 sequences were similar to any GenBank [19] sequence. Rare alleles with a frequency <5% were detected at most loci.

The 22 novel developed *M. coruscus* microsatellite markers vary widely in their degree of polymorphism. In total, 381 alleles were observed for the 22 loci; the number of alleles per locus varied from six at KMc16 to 24 at KMc1 and KMc4 (mean 17.32; Table 1). The observed heterozygosity ranged from 0.10 at KMc16 to 0.90 at KMc3 (mean 0.67), whereas the expected heterozygosity varied from 0.69 at KMc16 to 0.96 at KMc4 (mean 0.90; Table 1). The polymorphic information content (PIC) ranged from 0.65 to 0.95 (mean 0.87), revealing high information content. The development of powerful and efficient microsatellite markers with a high PIC (>0.5) is an essential step in the analysis of the genetic background of the endangered *M. coruscus*. In this study, all newly developed 22 polymorphic loci were confirmed to have a high degree of polymorphism (mean PIC = 0.87), enabling further resolution of the population structure and other genetic studies [20].

There was no evidence of genotyping errors or allele dropouts due to stuttering that affected the allele scoring. Samples that failed to amplify after the rerun were excluded, and thus the likelihood that poor DNA quality affected the results was low. The MICRO-CHECKER analysis revealed that some loci could have been influenced by one or more null alleles in the samples tested; our data demonstrated that 14 loci (KMc1, KMc2, KMc4, KMc5, KMc8, KMc9, KMc10, KMc12, KMc16, KMc17, KMc18, KMc19, KMc20 and KMc21) were affected. Deviation from Hardy–Weinberg equilibrium (HWE) (*p* < 0.002) was evident at 14 loci, which included KMc7 and 13 of the loci having null alleles (all except KMc8), indicating that deviations from HWE were due to heterozygote deficiency. The deficiency in heterozygotes is unlikely to be a technical artifact, as it was observed for the majority of markers. The existence of null alleles is regarded as the most likely cause, because null alleles are widely observed in other mollusks [7,21,22]. The high frequency of null alleles in microsatellite is thought to be due to an extremely high level of polymorphism in the flanking regions that are targeted by the PCR primers [7]. Furthermore, in recent years, evidence from genome sequencing and mass EST data analysis of mollusks showed a frequency of one SNP every dozens to hundred of base pairs [23,24]. Indeed, null alleles were observed for 14 loci in our study. Further investigations are required to determine whether these null alleles are due to population subdivision (a Wahlund effect) or inbreeding. Regardless, the possibility of the high frequency of nulls by the relatively small number of samples collected and tested cannot be excluded. Examination of linkage disequilibrium for all pairs of loci using a likelihood-ratio test by ARLEQUIN version 3.0 [25] revealed that twelve loci observed in *M. coruscus* were in linkage equilibrium (*p* < 0.05) (Table 1). These markers should be used with caution. The observed linkage disequilibrium between loci could be due to sampling errors in such as small cohort.

Because this study was limited by the number of populations screened, the genetic diversity parameters and the HW disequilibrium in the natural samples might be explained by data from additional populations, which would provide more precise estimates for the genetic characterization of the microsatellite loci. Thus, our results should be interpreted with caution.

The genetic diversity of *M. coruscus* for the 22 polymorphic microsatellite loci identified in this study was high. Two leading indices, allelic diversity and heterozygosity, were considerably high (Table 1). A high level of genetic diversity seems to be a common characteristic of marine bivalves [8,26,27]. Molecular genetic diversity has been associated with life history traits that reflect habitat types [28]. A large population size and high nucleotide mutation rates may be the main contributors [7,29]. Although the genetic diversity of *M. coruscus* is relatively high, its populations are most likely declining [2]. This result indicates that *M. coruscus* may be well conserved in offshore areas after the loss of habitats resulting from competition with an invasive species, *M. galloprovincialis*, in the coastal areas of Korea. Thus, the genetic diversity of *M. coruscus* should be protected. Therefore, there is an urgent need to create effective management strategies for the conservation of natural populations of *M. coruscus*.

Additionally, the cross-species amplification of 22 microsatellite markers was performed in another congener species, *M. galloprovincialis*. The two species studied in this experiment are the most important mussel fishery resources in Korea. Generally, the number of amplified loci tends to decrease in proportion to the increasing divergence between species [30,31]. Although these species are closely related based on taxonomy, none of the primer pairs was effectively amplified the target sequences using the same PCR conditions. One drawback of SSRs is their high species-specificity, resulting in low cross-amplification success. Poor cross-species amplification of microsatellite DNA loci due to widespread null alleles because of high mutation rates has been reported for marine bivalves [7].

## 3. Experimental Section

### 3.1. Sample Collection and 454 Sequencing

Thirty samples of *M. coruscus* were collected from the offshore areas of Taean, Korea, in October 2009. For cross-amplification, 30 samples of *M. galloprovincialis* were collected from the coast near Tongyeong, Korea, in October 2012. The species identification was verified through morphological distinctions [1] and the comparison of partial COI (cytochrome oxidase subunit I) nucleotide sequences [17]. For microsatellite isolation, the TNES-urea buffer method [32] was used to isolate high-molecular-weight DNA (≥2 μg) from the mantle musculature tissue of an individual mussel of the species *M. coruscus*. A whole-genome shotgun library was generated from 2 μg of the genomic DNA with the GS DNA Library Preparation Kit (Roche Applied Science, Indianapolis, IN, USA) according to the manufacturer’s protocol. The DNA library was titrated by means of sequencing on the Genome Sequencer FLX system (Roche Applied Science, Indianapolis, IN, USA). Based on the results of the titration sequencing run, an appropriate amount of the DNA library was used for the emulsion PCR set-up. Subsequently, the clonally amplified DNA fragments bound to the capture beads were enriched and sequenced on a quarter-plate in a 454 Life Sciences Genome Sequencer FLX Titanium instrument (Roche Applied Science, Indianapolis, IN, USA). For genotyping to characterize the microsatellite DNA loci, total DNA from the mantle-clips of each sample was extracted using a MagExtractor-Genomic DNA Purification Kit (Toyobo, Osaka, Japan) for an automated DNA extraction system, the MagExtractor MFX–2100 (Toyobo, Osaka, Japan). The extracted genomic DNA was stored at −20 °C until further use.

### 3.2. Microsatellite Discovery and Primer Screening

The resulting raw sequences for *M. coruscus* were assembled into contigs using Newbler 2.3. A Perl script was performed to select sequences longer than 300 bp with a minimum of five repeats of di- to tetra-nucleotide repeat motifs. Of the reads identified, a subset that had a minimum of ten repeats was selected. For these reads, a PERL script was also used to design the primers, with the following criteria to identify loci with a good likelihood of reliable amplification: (i) GC content 30%–90%; (ii) product size 90–250 bp; (iii) primer length 18–20 bp; and (iv) melting temperature 58 °C–68 °C.

### 3.3. DNA Amplification and Genotyping

All of the newly designed PCR primer pairs were tested for consistency of the PCR amplification and polymorphisms, which was performed on a sample set from eight mussels collected from Taean, Korea. The PCR amplification was performed using an ABI 9700 Thermal Cycler System (Applied Biosystems) in a 25-μL reaction containing 12.5 μL of 2× Multiplex PCR Pre-Mix (SolGent, Korea; Cat. No. SMP01-P096), 100 ng of template DNA and 10 pmol of each primer, with the forward primer from each pair being 5′-end-labeled with the 6-FAM and HEX dyes (Applied Biosystems). The PCR reaction ran for 15 min at 95 °C, followed by 30 cycles of 20 s at 95 °C, 40 s at 54 °C, and 2 min at 72 °C, with a final extension of 3 min at 72 °C. The annealing temperature of 54 °C was 4 °C–5 °C below the Tm estimated from the nucleotide compositions of the primer pairs. If no amplification was detected, that primer was excluded from further analysis by multiplex PCR. The production of PCR products was analyzed based on the presence of a visible band after running 5 μL of the PCR product on a 5% denaturing agarose gel. The 1 kb Plus DNA Ladder molecular weight marker (SolGent, Korea; Cat. No. SDL54-B500) was used as a standard to assess the product size. Some loci could not be amplified for all or any of the samples or yielded faint bands, even after adjusting the PCR conditions. We excluded these loci from further testing. For the remaining loci, genetic variation was tested in a total of 30 individuals collected from Taean. Microsatellite polymorphisms were tested using an ABI PRISM 3100 Automated DNA Sequencer (Applied Biosystems), and alleles were designated by PCR product size relative to a molecular size marker (GENESCAN 400 HD [ROX], Applied Biosystems). The fluorescent DNA fragments were analyzed using the GENESCAN (ver. 3.7) and GENOTYPER (ver. 3.7) software packages (PE Applied Biosystems). The samples were multiplexed for genotyping by pooling samples tagged with different dyes within a well. We assessed the reliability of the primers by repeating the amplification and genotyping for 16 samples (53.3%).

Finally, all of the newly developed microsatellite loci (including poly- and monomorphic loci) in *M. coruscus* were assessed for cross-amplification in another congener species, *M. galloprovincialis*, using 30 individuals.

### 3.4. Genetic Analysis

MICRO-CHECKER version 2.2.3 [33] was used to detect genotyping errors due to null alleles, stuttering, or allele dropout using 1000 randomizations. To access genetic diversity, the number of alleles per locus (*N*_A_), the observed and expected heterozygosities (*H*_o_ and *H*_e_, respectively) and the polymorphic information content (the PIC is an indicator of the utility of the marker for linkage or population genetic studies) based on the allele frequencies pooled across all samples were determined using CERVUS version 3.03 [34]. Deviations from Hardy-Weinberg equilibrium (HWE) and linkage disequilibrium (LD) were determined using Arlequin version 3.0 [25].

## 4. Conclusions

In conclusion, we report, the development of 22 polymorphic microsatellite markers that will enable us to examine the historical and current genetic structural patterns and diversity of *M. coruscus* as a threatened species in Korea. This study is among the first few studies to demonstrate the next-generation sequencing method of microsatellite acquisition for a commercially important fishery species [35,36]. In the near future, the microsatellites described here will be used in studies on population genetics, conservation genetics, and the effective management of *M. coruscus*.

## Figures and Tables

**Table 1:** Characteristics of the 22 polymorphic microsatellite loci in *Mytilus coruscus* and cross-amplification in another congener species.

Locus	Repeat motif	Primer sequence (5′-3′) a		*T*_a_ (°C)	Population (*N* = 30)					Cross-amplification(*N* = 30)
		
		F	R		N_A_	*H*_o_	*H*_e_	PIC	GeneBank Accession No.	*M. galloprovincialis*
KMc1 ^†^	(AT)_11_	GCAGCTCTTACGTGTTGATC **6-fam**	ATACACGCATGTAGATGCAC	54	24	0.83 ^*^	0.95	0.94	JQ678740	F
KMc2 ^†^	(ATC)_30_	GGCTCAGTGTGTCAATCATC **hex**	TATGTAGGTTCTGCAAAGTGG	54	19	0.71 ^*^	0.93	0.91	JQ678741	F
KMc3	(TGA)_12_	ATGTTCGAACTGCGCTGATG **6-fam**	CGCTCAAACTAGTTGGGTTA	54	18	0.90	0.93	0.91	JQ678742	F
KMc4 ^†^	(TA)_13_	CTGCGATGCTTTCAGATGTC **hex**	AAGCTTAAAATAGCCCGCGA	54	24	0.47 ^*^	0.96	0.94	JQ678743	F
KMc5	(GATT)_17_	CCTAGTGTCTCTCTAGTCCA **hex**	CATAAGGAATTTCCAGCCACA	54	14	0.59 ^*^	0.91	0.88	JQ678744	F
KMc6	(AC)_13_	CCACATCAAGTGAGAGAGAG **6-fam**	GACAAATGTAGTGAACGACG	54	12	0.70	0.84	0.81	JQ678745	F
KMc7	(TCAA)_13_	ACGTAGCGTGAAACCTTCAC **6-fam**	CTATGCAAATCACGTTGCTG	54	20	0.89 ^*^	0.95	0.93	JQ678746	F
KMc8	(TA)_11_	CCCGATGGAGAAAGTTTGTC **hex**	ATATGATCCATCGCCCCGTA	54	10	0.63	0.83	0.79	JQ678747	F
KMc9	(TA)_11_	CGGTGTGGGAAGAACGTAAA **6-fam**	ATTCGTGCATATTCAGGACG	54	17	0.76 ^*^	0.92	0.90	JQ678748	F
KMc10 ^†^	(GAT)_19_	ATGACGGGTAGAACCTGACA **hex**	CTCAATGTGTCGGTCTAGTA	54	19	0.53 ^*^	0.95	0.93	JQ678749	F
KMc11	(ATCA)_12_	AGGGGCTGTTAAGACTGTCG **hex**	ATTCCACAGTCATTGGTCC	54	19	0.89	0.93	0.90	JQ678750	F
KMc12	(TA)_12_	GTTAAGTGCACACCTGTGAG **hex**	AATTCACCAGGAGCATTGTG	54	13	0.22 ^*^	0.76	0.73	JQ678751	F
KMc13	(GAT)_22_	TGGAAGTGTGTACTGGGCTA **6-fam**	AAGAAATGGAACAGGAGCAG	54	21	0.87	0.95	0.93	JQ678752	F
KMc14	(CAT)_12_	AAACATTTTGCCGCTGGACG **hex**	ATGCTTCCCCAACTTGTTAC	54	22	0.90	0.93	0.91	JQ678753	F
KMc15	(ATT)_16_	GAGGGCCTTAGGGAAGATTA **hex**	ACGTCTATCAACCTCAGAAG	54	21	0.87	0.93	0.91	JQ678754	F
KMc16 ^†^	(TG)_12_	CCCTACACTCGGACTTTACA **6-fam**	CCGTTACGAACGATTACTAG	54	6	0.10 ^*^	0.69	0.65	JQ678755	F
KMc17 ^†^	(ATA)_11_	GATCACCCTGTTTCAGAGTC **6-fam**	ATTGTATATGAGGGCCTCAG	54	12	0.57 ^*^	0.89	0.86	JQ678756	F
KMc18 ^†^	(AT)_14_	GCCCAAACGACGTGTATTCA **6-fam**	GTATGATCCATCGCCCCGTA	54	18	0.31 ^*^	0.88	0.86	JQ678757	F
KMc19 ^†^	(AG)_16_	GGTCGTGTCCAAAGGAATTG **6-fam**	CTAAATAACTGCACGACTCG	54	21	0.73 ^*^	0.95	0.93	JQ678758	F
KMc20 ^†^	(AT)_12_	TGATCCTTTCACACAGCAGG **hex**	CTTCTCGTGCTCCATGTACA	54	13	0.87 ^*^	0.90	0.87	JQ678759	F
KMc21 ^†^	(TG)_11_	GGGTCAGATGACTCTGGAAA **hex**	CTTTATGGCGTGTCAAATCG	54	22	0.63 ^*^	0.89	0.87	JQ678760	F
KMc22	(AG)_11_	GGCAATCATACCACATCACC **hex**	CGGTATGTGCTGCCCTTTTA	54	16	0.73	0.89	0.86	JQ678761	F

Locus ^†^ is in linkage disequilibrium at one or more loci with one another;

a Primers were 5′ end labeled with the indicated dyes; The optimal annealing temperature (*T*_a_), the number of samples (*N*), the number of alleles per locus (*N*_A_), expected heterozygosity (*H*_e_), observed heterozygosity (*H*_o_) and the polymorphism information content (PIC) are given for each locus. Exact tests of Hardy–Weinberg equilibrium showed significant heterozygote deviation (^*^ *p* < 0.002). Calculations assume that individuals with one microsatellite band are homozygous for the allele; “F” means “failed to amplify or multiple non-specific amplification”.
